# Cancer Risk and Mutational Patterns Following Organ Transplantation

**DOI:** 10.3389/fcell.2022.956334

**Published:** 2022-06-28

**Authors:** Yangyang Shen, Di Lian, Kai Shi, Yuefeng Gao, Xiaoxiang Hu, Kun Yu, Qian Zhao, Chungang Feng

**Affiliations:** ^1^ College of Animal Science and Technology, Nanjing Agricultural University, Nanjing, China; ^2^ State Key Laboratory of Agrobiotechnology, College of Biological Sciences, China Agricultural University, Beijing, China; ^3^ College of Applied Engineering, Henan University of Science and Technology, Sanmenxia, China; ^4^ Sanmenxia Polytechnic, Sanmenxia, China; ^5^ College of Animal Science and Technology, China Agricultural University, Beijing, China

**Keywords:** organ transplantation, cancer, next-generation sequencing technology, mutational patterns, organoids

## Abstract

The rapid development of medical technology and widespread application of immunosuppressive drugs have improved the success rate of organ transplantation significantly. However, the use of immunosuppressive agents increases the frequency of malignancy greatly. With the prospect of “precision medicine” for tumors and development of next-generation sequencing technology, more attention has been paid to the application of high-throughput sequencing technology in clinical oncology research, which is mainly applied to the early diagnosis of tumors and analysis of tumor-related genes. All generations of cancers carry somatic mutations, meanwhile, significant differences were observed in mutational signatures across tumors. Systematic sequencing of cancer genomes from patients after organ transplantation can reveal DNA damage and repair processes in exposed cancer cells and their precursors. In this review, we summarize the application of high-throughput sequencing and organoids in the field of organ transplantation, the mutational patterns of cancer genomes, and propose a new research strategy for understanding the mechanism of cancer following organ transplantation.

## 1 Introduction

Organ transplantation is one of the greatest achievements of modern medicine. For patients with advanced disease, solid organ transplantation is a helpless but hopeful option (Bezinover and Saneret, 2019). However, even if the organ donor does not carry cancer, the cancer rate is higher in patients who have undergone organ transplants than in those who have not been transplanted. In other words, it seems that this surgery itself has the side effect of increasing cancer rates (Matser et al., 2018). Although the application of immunologic drugs improves the survival of solid organ recipients, it is still an important cause of *de novo* neoplastic malignancies following organ transplantation. We know that almost all cancers are caused by DNA mutations. Using next-generation sequencing technology, researchers found that these *de novo* somatic mutations formed a “molecular fingerprint” with mutational signatures in the tumor genome (Petljak et al., 2019). From this perspective, the following five aspects can be studied to characterize the mutational process of tumor genomes after organ transplantation: observed mutation types, local sequence background, distribution of mutations across the whole genome, evidence of DNA repair, and timing of cancer evolution (Martincorena and Campbell, 2015).

Therefore, we reviewed cancer risk following organ transplantation from the following aspects: clinical application of next-generation sequencing technology, application of organoid technology, and mutational characteristics of tumor genomes. We focused on the elaboration of the mutational signatures in tumor genomes, which has been a research hotspot in recent years, and we took this as a starting point to propose a new research strategy for understanding the mechanism of cancer following organ transplantation.

## 2 Cancer Risk and Organ Transplantation

**FIGURE 1:**
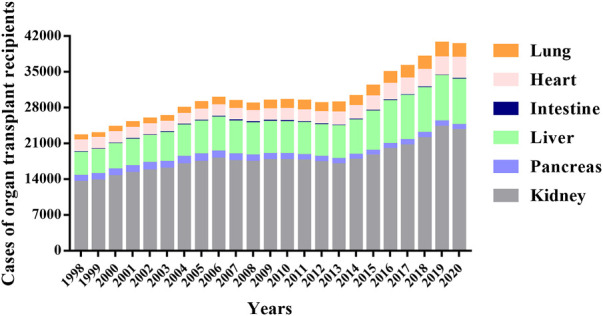
Total cases of different organ transplants per year from 1998 to 2020. The data were obtained from the Scientific Registry of Transplant Recipients (SRTR) website (https://srtr.transplant.hrsa.gov/), which provides the current status of solid organ transplantation in the United States for researchers interested in studying all aspects of solid organ transplantation.

Organ transplantation provides a life-saving treatment for patients with serious organ diseases (Noone et al., 2022). More than 100,000 patients worldwide receive organ transplants each year, which are conducted widely to people of all ages (Sherston et al., 2014). The Scientific Registry of Transplant Recipients (SRTR) report showed that the number of organ transplants performed annually has shown a steady upward trend over the past few decades and peaked in 2019, with a decline in 2020 likely due to the COVID-19 pandemic **(** Figure 1 and Supplementary Table S1 **)**. In 2020, a record number of solid organ transplants were performed in the U.S., which including 23,642 kidney, 962 pancreas, 8,906 liver, 91 intestine, 4,180 heart, and 2,633 lung transplants. Compared with the year 2010, kidney transplants (+39.9%), liver transplants (+41.6%), heart transplants (+59.2%), and lung transplants (+46.7%) increased significantly. Over the same period, pancreatic and intestinal transplants dropped 21% and 49%, respectively. The demand for transplants continues to rise (Colvin et al., 2022; Horslen et al., 2022; Israni, 2022; Kandaswamy et al., 2022; Kwong et al., 2022; Lentine et al., 2022; Valapour et al., 2022).

Nevertheless, malignancy is known as one of the three major causes of death in patients following organ transplantation, especially those in the last stage of cancer, and is, therefore, of increasing concern to many scientists (Chapman et al., 2013). Previous studies demonstrated that immunosuppression is a major risk factor for tumorigenesis in transplant recipients. Long-term immunodeficiency may increase the risk of infection-related malignancies, such as EBV (EB virus), HBV (Hepatitis B virus), HCV (Hepatitis C virus), human papilloma virus (HPV), HHV-8, HTLV-1, HIV and *Helicobacter pylori* (Adami et al., 2003). In addition, the impaired immune detection function of tumor cells and the use of immunosuppressive agents are both key factors that induce tumors in organ recipients directly (Hunt, 2006).

Overall, multiple studies have shown that organ transplant patients had a significantly higher risk of malignancy than the general population. The risk of cancer was affected mainly by the type of transplant, which depended on three factors: use of immunosuppressive drugs, underlying medical comorbidities, and end-stage organ disease (Charlton et al., 2018). The most common cancer among transplant recipients was non-Hodgkin’s lymphoma (14% of all cancers in transplant recipients), lung cancer (13%), liver cancer (9%), and kidney cancer (7%) (Engels et al., 2011; Sargen et al., 2022). Compared with the general population, the risk of malignant tumors after kidney transplantation was higher, and there was a high incidence of certain types of tumors (Villeneuve et al., 2007; Au et al., 2018; Musquera et al., 2021; Rodriguez-Aguilar et al., 2021). The incidence of malignant tumors varied from region to region. In the United Kingdom, lymphoma and renal cell carcinoma were common. In Australia, the incidence of skin cancer was very high. These differences in frequency of types of tumors in different regions may be related to the differences in the immunosuppressants demand of different races and life styles (Cheung et al., 2012; Cheung et al., 2014; Cheung and Tang, 2019).

The increased risk of malignancy in the organ transplant population was associated typically with viral infections, such as non-Hodgkin’s lymphoma, liver cancer, cervical cancer, and other viral infection-associated tumors (Engels et al., 2008; Pierangeli et al., 2015; Hernandez-Ramirez et al., 2017; Hortlund et al., 2017). In addition to the usual cancer, Monica et al. examined 694 distinct cancer subtypes, in which 33 manifested standardized incidence ratios that were elevated significantly. They identified specific rare cancers that were overrepresented in the population of solid organ transplant recipients (SOTRs). Some of these cancers may have increased as a result of immunosuppression and loss of immunosurveillance. They also held the view that assessing risk of rare cancers in SOTRs may provide etiological clues for other cancers that were linked to immunocompromised and viral infections (D'Arcy et al., 2021). Understanding the cancer risk characteristics of different solid organ transplant recipients contributes to early diagnosis, evaluation and treatment of post-transplant malignancies (Huo et al., 2020). At present, the treatment of malignant tumor after organ transplantation is still based on surgery, supplemented by chemotherapy, radiotherapy, endocrine therapy, and other comprehensive treatment. Only with the joint participation of a variety of disciplines and the development of personalized treatment plans will it be possible to improve the effectiveness of treatments for patients.

## 3 Clinical Application of Next-Generation Sequencing Technology

Next-generation sequencing technology (NGS), which is also known as large-scale parallel sequencing and high-throughput sequencing, mainly includes Roche, Illumina, MGI, and other platforms. Because of the existence of the most accurate molecular biology information, the simplified workflow, and lower cost, NGS technology has become a leader in molecular biology diagnostics (Behjati and Tarpey, 2013; Slatko et al., 2018). Based on the new generation of molecular diagnostic detection techniques developed by NGS technology, researchers can realize the early diagnosis of disease, and adapt a more rational treatment plan to achieve precision and personalized medical care (Morganti et al., 2019).

### 3.1 Next-Generation Sequencing Technology and Cancer Detection

Cancer is a genome-level disease in which each person is born with a unique genome that determines their risk of developing cancer. When the disease is triggered, it causes unique mutations in the genome quickly. Traditional diagnostic methods sometimes fail to identify cancer accurately because specimens are often mixed with multiple cell types and normal tissue. The clinical application of NGS in gene diagnosis of cancer is embodied mainly in individualized drug use, early diagnosis, and prediction of cancer risk. NGS technology analyzes genomic mutations with single-base resolution and provides comprehensive information for molecular diagnosis of tumors by measuring expression levels, splicing variations, non-coding RNA, DNA methylation, and protein-nucleic acid interactions (Zhao, 2019; Rodriguez-Martin et al., 2020; Xu and Richard, 2021; Papanicolau-Sengos and Aldape, 2022). The Cancer Genome Atlas (TCGA) and catalogue of cancer somatic mutations were established internationally through years of molecular biology research on cancer (Donehower et al., 2019; Linehan and Ricketts, 2019; Travaglino et al., 2020). Therefore, information about the genetic susceptibility of an individual can be obtained by detecting risk markers in the genome. In addition, during cancer treatment, a large number of genomic rearrangements and somatic mutations accumulate, which leads to increased risk of metastasis or drug resistance. The likelihood of recurrence and drug resistance can be determined by several genetic tests for prognostic markers, which provides a basis for targeted drug use and personalized cancer diagnosis.

The rapid development of methylation sequencing technology has greatly promoted the in-depth study of tumor pathogenesis. DNA methylation exists widely in the life process and is involved in the production of various diseases, especially in cancerization, so it has become an important biomarker for early detection of tumors (Luo et al., 2021; Galbraith and Snuderl, 2022). Guo et al. used the dual signals of cancer markers and tissue-specific CpG methylation patterns to detect and trace the specific location of the tumor (Guo et al., 2017). Gai et al. attempted to use methylation “fingerprints” to identify cancer mutations directly from blood DNA (Gai et al., 2021). Although fewer mutations were found, the liver was correctly identified as the source of tumor-derived molecules. Hence, the methylation modification of cell DNA can be used as a biomarker in many clinical fields such as non-invasive prenatal testing, early cancer screening, early diagnosis, and organ transplantation evaluation (Muller and Gyorffy, 2022; Zeng et al., 2022).

Furthermore, the cellular heterogeneity of tumors is a major obstacle to understanding and treating oncology. Single-cell sequencing technology can make up for limitations of traditional high-throughput sequencing by sequencing single cells, so as to reveal cancer gene structure and expression more accurately from the cellular level, and can be used to reflect the heterogeneity among tumor cells (Rantalainen, 2018; Malone and Humphreys, 2019; Natarajan et al., 2019; Winterhoff et al., 2019; Ung et al., 2022). Li et al. mapped the heterogeneity of immune cells after liver transplantation for the first time by single-cell sequencing and other techniques. T cells and myeloid cells showed different tissue distribution and function in human liver and peripheral blood (Li et al., 2022). Yang et al. sequenced about 20,000 cells of normal and steatotic donor liver tissue after transplantation by single-cell sequencing technology and drew the first single-cell map of steatotic transplanted liver (Yang et al., 2021). This study was launched primarily to explain the important mechanism of steatotic donor liver in the process of transplantation injury from the single-cell transcriptome level. The combination of single-cell sequencing and high-throughput sequencing technology makes it possible to analyze the heterogeneous molecular characteristics of tumor cells, which is of great significance for discovering early-stage tumor cells and guiding targeted precision therapy (Gupta and Kuznicki, 2020; Liu et al., 2020; Malone et al., 2020; Varma et al., 2021).

### 3.2 Clinical Demands for Cancer Diagnosis

Urgent demands still exist in the oncology field, especially for the treatment of advanced tumors and screening for early tumors. NGS can detect multi-gene and multi-locus mutations simultaneously, changes in gene copy number, fusion genes, oligo nucleation and deletion mutations (Sabour et al., 2017). In addition, NGS can also diagnose the Microsatellite Instability (MSI) status of specimens and Tumor Mutation Burden (TMB), which has significant advantages over traditional techniques (Samstein et al., 2019). The stages of cancer diagnosis and treatment may occur during a person’s life from birth to death (Bray et al., 2021). GLOBOCAN 2020 Global Cancer Statistics estimated that there were19,292,789 new cancers cases worldwide (Freddie et al., 2018). The top 10 cancer types accounted for more than 60% of all new cancers. Using these published data from GLOBOCAN 2020, Xia et al. estimated that there will be approximately 4,820,000 and 2,370,000 new cancer cases, and 3,210,000 and 640,000 cancer deaths in China and the U.S. in 2022, respectively (Xia et al., 2022). There are significant differences in the burden of cancer worldwide, but the situation of cancer prevention and control in all countries is serious. Effective and locally tailored cancer detection and control measures are essential to reduce the global burden of cancer in the future. At present, the industry and academia are actively developing the early screening technology of tumors based on NGS liquid biopsy, which is expected to be applied to the early and even ultra-early screening of tumors in the future (Chen and Zhao, 2019).

## 4 Mutational Patterns of the Cancer Genome

Mutation, which is an important source of species diversity, is the driving force of biological evolution (Lynch, 2010a; Keightley, 2012; Lynch et al., 2016). However, the accumulation of mutations, that lead to the development of cancer, also accompany the aging of life. The PCAWG (Pan-Cancer Analysis of Whole Genomes) consortium has identified common genome-wide mutation patterns in more than 2,600 cancers and published several articles in the journal *Nature*. There papers cover some aspects of tumor-driven mutations, non-coding regions, mutation characteristics, structural variations, tumor evolution, and RNA changes, which are crucial to understanding the full genetic complexity of cancer (Islamovic et al., 2014; Alexandrov et al., 2020; Calabrese et al., 2020; Li et al., 2020; Mosher et al., 2020; Rheinbay et al., 2020).

### 4.1 Mutational Processes in Cancer

Somatic and germline mutations accumulate in every mammalian individual during their lifetime (Lindsay et al., 2019). Somatic mutations may increase the risk of cancer, and germline cell mutations are passed along to the next generation and have important implications for the evolutionary inheritance of the entire population (Li et al., 2021). During ontogenesis, somatic and germline cells are derived from the same zygote, undergo multiple rounds of DNA replication and mitosis, and share the same DNA replication and repair mechanism. Nevertheless, the two types of cells differ in their response to mutations and also show differences in mutation rates. The mutation rate of germline cells is much lower than somatic cells, which is generally considered by research (Milholland et al., 2017; Moore et al., 2021). A highly accurate DNA replication system exists in eukaryotic cells, which include nucleotide selection, proofreading, and timely repair of mismatches to ensure that mutations are kept at an extremely low level (Bailly and Gartner, 2013). Nonetheless, the accumulation of mutations in mammals over a lifetime still represents a considerable amount, given that many organisms have large genomes and undergo many replications during ontogeny. Although the mutation rate is low, the occurrence of mutation is not a small probability event for an organism (Frank, 2014).

All cancers are caused by somatic mutations (Alexandrov et al., 2013). Both somatic mutations that accumulate during disease development and germline mutations inherited from parents are present in typical tumors (Stratton et al., 2009; Dasari et al., 2021). At the same time, the cell-selective advantage of tumor cells for preferential growth or survival enables them to accumulate mutations rapidly in a short time. However, mutations can also occur in some normal tissues of the human body, because the older the individual is, the more cell divisions he has undergone, especially those tissues that are capable of cell sorting and proliferation (Conrad et al., 2011; Blokzijl et al., 2016; Martincorena et al., 2018). There is a strong correlation between organ tissues and the number of cell divisions that are at high risk of developing cancer in humans (Kamalabadi-Farahani and Kia, 2020). Certainly, spontaneous mutations are also present in cells that do not proliferate. These mutations are caused by the failure to repair properly during DNA repair, not during DNA replication. Broadly speaking, mutations arise from replication errors or from DNA damage that is either repaired incorrectly or left unrepaired.

DNA damage can be caused by exogenous factors, which include chemicals, ultraviolet (UV) light, and ionizing radiation (Pfeifer et al., 2005), by endogenous factors, such as reactive oxygen species, aldehydes, or mitotic errors, or by enzymes involved in DNA repair or genome editing, among others (Lehmann, 2006; Stephens et al., 2009; Lynch, 2010b). Additionally, viruses and endogenous retrotransposons cause insertions into the DNA sequence. Most cancers carry about 1000–20,000 individual somatic mutations and hundreds of Indels and structural rearrangements. The mutation rate varies among cancers. For example: the mutation rate is lowest in childhood brain tumors and highest in lung (smoking) and skin cancers (UV radiation), which are also the most common cancers following organ transplantation (Alexandrov et al., 2013).

### 4.2 Mutational Spectrum

Different mutational processes have different mutational characteristics. Unique mutational patterns can help us to identify mutational processes in existing or somatic mutations and to quantify their role in cancer development. Single nucleotide mutations (SNMs) can be classified as transition (purine to purine, pyrimidine to pyrimidine) or transversion (a substitution between pyrimidine and purine). In general, transition is less likely than transversion to cause changes in amino acid sequences. The types of substitutions between bases form the mutational spectrum. In the human spectrum, transitions were more frequent than transversions, which accounted for 60% of the spectrum (Goldmann et al., 2016; Wong et al., 2016). The most common conversion type is C:G > T:A, the cause may be spontaneous deamination of 5-methyl cytosine near the CpG site. 5-methylcytosine and cytosine are deaminated easily in mammalian DNA to form thymine and uracil, which results in a G:T mismatch or G:U mismatch, this can be repaired to A:T (Kong et al., 2012). Several studies have shown that although CpG sites make up only about 1% of the human genome, C:G > T:A conversions accounted for 40% of the total point mutations. Transversions are relatively rare in the human mutational spectrum, 50% less than conversions. Among them, the formation of 8-oxyguanine leads to the cause of G:C > T:A transversion. Because oxidized guanine can form an 8-OXOG:A pair, this leads to the G to T mutation (Nakabeppu et al., 2006). Mutations are distributed unevenly across the genome and correlated with the state of the local chromatin. In the tumor genome, somatic mutations are more frequent in late-replicating regions.

**FIGURE 2:**
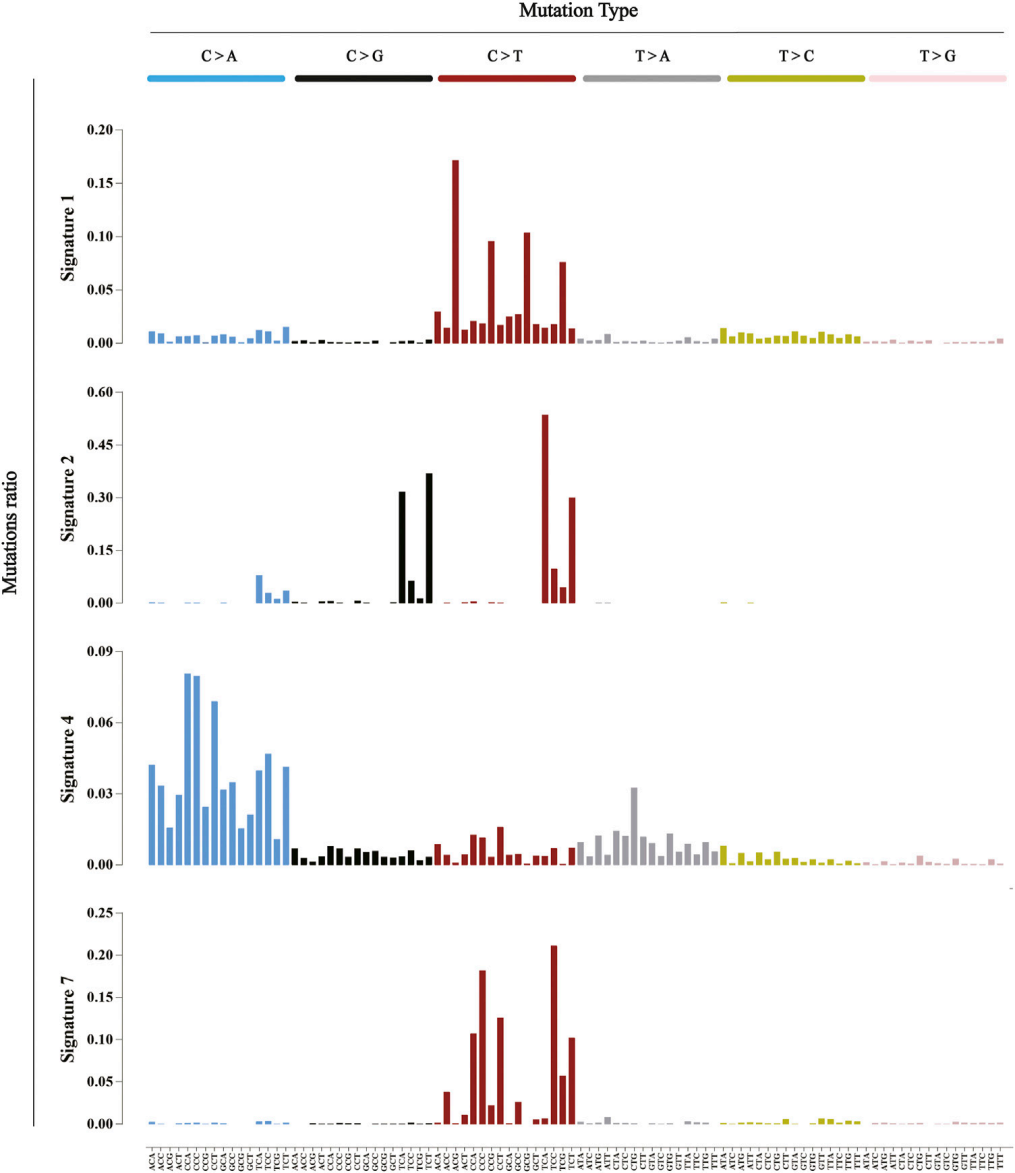
Five mutational signatures found in human cancer. The six alternate probability bars are shown in different colors. Mutation types are on the horizontal axis, while the vertical axis depicts the percentage of mutations attributed to a particular mutation type. These five signatures are analyzed from the COSMIC database (http://cancer.sanger.ac.uk/cosmic/signatures).

Mutational signatures provide an additional dimension to the interpretation of cancer genomes, and they summarize the biology of the tumor from each patient. There was substantial variation among the cancers in numbers of the six classes of base substitutions: C > A, C > G, C > T, T > A, T > C, and T > G (all substitutions are referred to by the pyrimidine of the mutated Watson-Crick base pair). By considering the immediate 5′ and 3′ of each mutated base, combining the sequence context in which the mutation occurred can provide greater insight into the mutational process at work. There are 96 possible mutated trinucleotides (six classes of base substitutions and 16 possible sequence contexts for each mutated base), which are particularly useful for distinguishing mutational signatures that cause the same substitutions but in different sequence contexts (Alexandrov et al., 2020). The COSMIC catalogue revealed several distinct, validated mutational signatures by applying this approach to most types of cancer. For examples, Signatures one was characterized by the prominence of C > T substitutions at NpCpG trinucleotides, which was probably related to the relatively elevated rate of spontaneous deamination of 5-methyl-cytosine. Signature two was characterized by C > T and C > G mutations at TpCpN trinucleotides, that converted cytidine to uracil, which was due to the base excision repair and DNA replication activity of the APOBEC family of cytidine deaminases (Roberts et al., 2013). In addition to these two examples of endogenous mutational processes, there were features that were ascribed to exogenous factor exposure. For example, Signature four was found in lung cancer and was associated with carcinogens, such as the tobacco. Signature seven was found in malignant melanoma and squamous carcinoma of the head and neck, with an ultraviolet-light-induced mutational signature **(** Figure 2)**.** This research also observed many different combinations of signatures and most individual cancer genomes exhibited more than one mutational signature. At least two mutational signatures were observed in most cancer classes, and up to six were observed in liver, uterine, and gastric cancers. Although some of these differences may be due to differences in the ability to extract signatures, it seems that some cancers have a more complex mutation process than others.

## 5 Application of Organoids in Organ Transplantation

An organoid is a novel,three-dimensional, multicellular aggregate derived from stem cells or organ progenitors; this enables them to self-renew and to self-organize continuously, and to then form tissues similar to the original organs in structure and function (Bleijs et al., 2019; Corro et al., 2020; Schutgens and Clevers, 2020). According to the cell origin, organoids can be classified into Adult Stem Cells (ASC), Pluripotent Stem Cells (PSC), or Patient-derived Organoid organs (PDOs). PSC can be divided into Embryonic Stem Cells (ESC) and induced Pluripotent Stem Cells (iPSC) (Mora et al., 2017; Montel-Hagen et al., 2019; Mun et al., 2019; Takebe and Wells, 2019). Organoids from diverse sources lead to their different properties and applications. For example, brain organoids, which are derived from PSCs, are used mainly to study psychiatric genetic diseases. ASCs, which are derived from regenerative precursor cells in tissues, are used mainly in the study of adult tissue biology, tissue regeneration, and precision medicine.

### 5.1 Precision Medicine

In recent years, the concept of “precision medicine” has attracted widespread attention by the public, and the emergence of organoids has created unprecedented new opportunities for precision medicine in cancer. Tumor heterogeneity is one of the main reasons for ineffective or resistant recurrence of antitumor drugs. Using organoid culture technology, tumor organoids were established from patient-derived tumor tissue and tested *in vitro* for the efficacy of tumors after radiation, chemotherapy, and targeted drug therapy. Therefore, more and more studies have established organoid biobanks for high-throughput screening of antitumor drugs and prediction of drug responses.

Yan et al. established a primary gastric cancer organoid (GCO) biobank that comprised normal, dysplastic, cancer, and lymph node metastases (*n* = 63) from 34 patients, which also included detailed whole-exome and transcriptome analysis. They also tested and identified sensitivity of GC to some therapeutic agents in the clinical phase of development, which opened up new therapeutic opportunities (Yan et al., 2018). The innovative application of induced pluripotent stem cell (iPSC) technology and organoid technology to genetic neurometabolic diseases and for evaluation of individualized drug toxicity are conducive to elucidating new pathologic and therapeutic strategies for human diseases, which provides a platform for large-scale drug screening for personalized treatment of cancer. Using patient-derived iPSCs and differentiated hepatoid cells as models, Liu et al. elucidated the mechanism of Valproic Acid (VPA) -induced hepatotoxicity of Alpers-Huttenlocher syndrome (AHS), and they proposed screening strategies for candidate drugs (Li et al., 2015). Based on the same model, they also found that liver cells in patients with MDS (Mitochondrial DNA Depletion Syndrome) were more sensitive to iron death caused by iron deposition (Guo et al., 2021).

In recent years, emerging gene editing technology (CRISPR-Cas) has brought great changes to the study of human biology, and brought new hope for precision medicine as well (Zhan et al., 2019; Hendriks et al., 2021). As is well known, experimental science has benefited from the combination of CRISPR with *in vitro* scalable stem cell systems and their derived organoids (Liu et al., 2018; Lannagan et al., 2019; Geurts et al., 2020). Significant steps still need to be taken to improve the efficiency and safety of CRISPR-edited human stem cells while optimizing delivery/transplantation for *in vivo* purposes (Hendriks et al., 2020). Lo et al. utilized wild-type, human gastric organoids to establish the first forward, genetic, human ARID1A-deficient oncogenic transformation model using CRISPR/Cas9-engineered ARID1A depletion alongside a mutation of TP53, which is a co-occurring tumor suppressor. Coupled with a regulatory network-based analysis and high-throughput drug screening, they leveraged this human organoid model to discover potential mechanisms that underlay the role of ARID1A during oncogenic transformation of gastric epithelium (Lo et al., 2021). Therefore, the collaboration of CRISPR and organoid technology has produced a multifunctional toolbox to accelerate the research of human cancer genes, with important implications for precision medicine.

### 5.2 Regenerative Medicine

The application of organoids in the field of regenerative medicine is to transplant the organoids obtained from adult tissue stem cells into the body to repair damaged tissues, achieve no immunosuppression, and avoid huge costs for lifelong anti-rejection therapy (Nakamura and Sato, 2018; Edgar et al., 2020). With the development of various new organoids, organoid transplantation has been practiced in liver, extrahepatic biliary tract, lung, islet, brain, skin and other tissues and organs (Nikolic et al., 2017; Sampaziotis et al., 2017; Hu et al., 2018; Mansour et al., 2018; Yoshihara et al., 2020). Due to its unique proliferative ability and tissue specificity, organoid transplantation restored and repaired the structure of the body well without malformation or tumors (Lee et al., 2020). However, there is a serious shortage of donors and tissue rejection, so it is urgent to find new tissue sources (Takebe et al., 2013). Organoids can be amplified by homologous tissues for autologous transplantation and provide renewable resources for organ replacement strategies (Rossi et al., 2018).

Several studies have shown the prospect and unique advantages of organoids in regenerative medicine. Lebreton et al. demonstrated that the integration of hAECs (humanamniotic epithelial cells) into islet cell organoids has great potential in the development of cell-based therapies for type 1 diabetes (Lebreton et al., 2019). Sugimoto et al. generated a functional small intestinalized colon (SIC) by replacing the native colonic epithelium with ileum-derived organoids, which provided proof of principle for the use of intestinal organoids for regenerative purposes, and they offered a feasible strategy for SBS treatment (Sugimoto et al., 2019). By transplanting biliary organs *in vitro* into human livers under *in vitro* conditions, Sampaziotis et al. provided proof of principle that cholangiocyte organoids can be used to repair human biliary epithelium (Sampaziotis et al., 2021). For the first time, they confirmed that the use of organoids grown in the laboratory can be transplanted and function as human organs, which opened up a new way of human-derived organoid transplantation that laid the foundation for the clinical application of organoid transplantation.

## 6 Summary and Prospects

**FIGURE 3:**
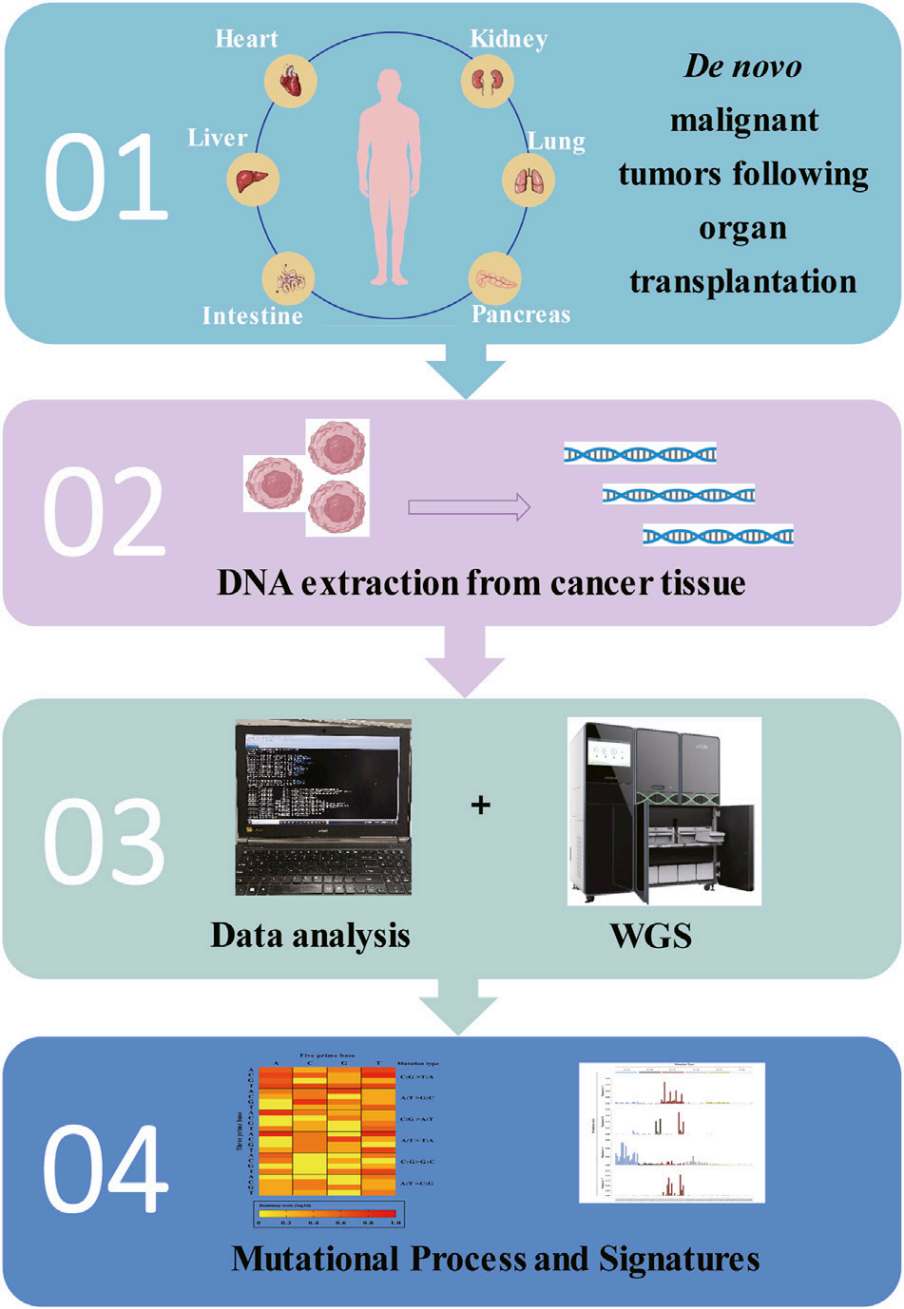
A pipeline for exploring the mutational mechanism of *de novo* malignant tumors following organ transplantation, combining current scientific research base.

The relentless efforts of scientists are aimed at conquering human diseases, alleviating the suffering of patients, and prolonging their lives. Organ transplantation appears to be a ‘double-edged sword’; it offers hope to patients with advanced disease, but the use of immunosuppressive agents increases cancer incidence in solid organ recipients. The development of organoid models over the past decade has been one of the major breakthroughs in the field of stem cells. Organoid models of diseases that are highly simulated, such as malignancies, are expected to continue to make new advances in precision medicine and regenerative medicine. Combined with biological 3D printing, functional therapy based on organoids should be realized. Combined with Human Cell Atlas technology, the organoid cell spectrum will advance disease-centered research, which includes rare genetic diseases, complex multifactorial diseases, and precision tumor therapy. Cancer genome sequencing has revolutionized scientists’ understanding of cancer genetics. Sequencing of the genomes of *de novo* malignancies in post-transplant patients will continue to generate cancer genes and mutational signatures that were unrecognized previously (Figure 3). Whole genome sequencing, novel statistical methods, and innovative mathematical models will help us answer these questions. Using detailed clinical information, these newly discovered signatures can be combined with treatment and clinical responses to help identify the driver genes for cancer development and to develop targeted drugs that will benefit patients. In the era of the COVID-19 pandemic, people all over the world are facing severe challenges. Using next-generation sequencing techniques, we will see systematic analysis of the genome of *de novo* malignancies following organ transplantation, which will provide a systematic analytical perspective on the mutational processes of human cancer development in the near future.
